# Sphingosine phosphate lyase insufficiency syndrome as a primary immunodeficiency state

**DOI:** 10.1016/j.jbior.2024.101058

**Published:** 2024-10-22

**Authors:** Saber Gharagozlou, NicolaA.M. Wright, Luis Murguia-Favela, Juliette Eshleman, Julian Midgley, Seha Saygili, Georgie Mathew, Harry Lesmana, Nadia Makkoukdji, Melissa Gans, Julie D. Saba

**Affiliations:** a Department of Pediatrics, University of California San Francisco, San Francisco, CA, USA; b Department of Pediatrics, Cummings School of Medicine, University of Calgary, Alberta, Canada; c Department of Pediatric Nephrology, Istanbul University-Cerrahpasa, Cerrahpasa Faculty of Medicine, Istanbul, Turkey; d Division of Pediatric Nephrology, Christian Medical College, Vellore, India; e Department of Medical Genetics and Genomics, Department of Pediatric Hematology/Oncology and BMT, Cleveland Clinic, Cleveland, OH, USA; f Department of Pediatrics, Division of Allergy & Immunology University of Miami Miller School of Medicine/Jackson Memorial Hospital, Miami, FL, USA

**Keywords:** Sphingosine phosphate lyase, *SGPL1*, SPLIS, Sphingosine-1-phosphate, Lymphopenia, Immunodeficiency, Infection

## Abstract

Sphingosine phosphate lyase insufficiency syndrome (SPLIS) is a genetic disease associated with renal, endocrine, neurological, skin and immune defects. SPLIS is caused by inactivating mutations in *SGPL1*, which encodes sphingosine phosphate lyase (SPL). SPL catalyzes the irreversible degradation of the bioactive sphingolipid sphingosine-1-phosphate (S1P), a key regulator of lymphocyte egress. The SPL reaction represents the only exit point of sphingolipid metabolism, and SPL insufficiency causes widespread sphingolipid derangements that could additionally contribute to immunodeficiency. Herein, we review SPLIS, the sphingolipid metabolic pathway, and various roles sphingolipids play in immunity. We then explore SPLIS-related immunodeficiency by analyzing data available in the published literature supplemented by medical record reviews in ten SPLIS children. We found 93% of evaluable SPLIS patients had documented evidence of immunodeficiency. Many of the remainder of cases were unevaluable due to lack of available immunological data. Most commonly, SPLIS patients exhibited lymphopenia and T cell-specific lymphopenia, consistent with the established role of the S1P/S1P1/SPL axis in lymphocyte egress. However, low B and NK cell counts, hypogammaglobulinemia, and opportunistic infections with bacterial, viral and fungal pathogens were observed. Diminished responses to childhood vaccinations were less frequently observed. Screening blood tests quantifying recent thymic emigrants identified some lymphopenic SPLIS patients in the newborn period. Lymphopenia has been reported to improve after cofactor supplementation in some SPLIS patients, indicating upregulation of SPL activity. A variety of treatments including immunoglobulin replacement, prophylactic antimicrobials and special preparation of blood products prior to transfusion have been employed in SPLIS. The diverse immune consequences in SPLIS patients suggest that aberrant S1P signaling may not fully explain the extent of immunodeficiency. Further study will be required to fully elucidate the complex mechanisms underlying SPLIS immunodeficiency and determine the most effective prophylaxis against infection.

## 1. Introduction

**Fig. 1.**
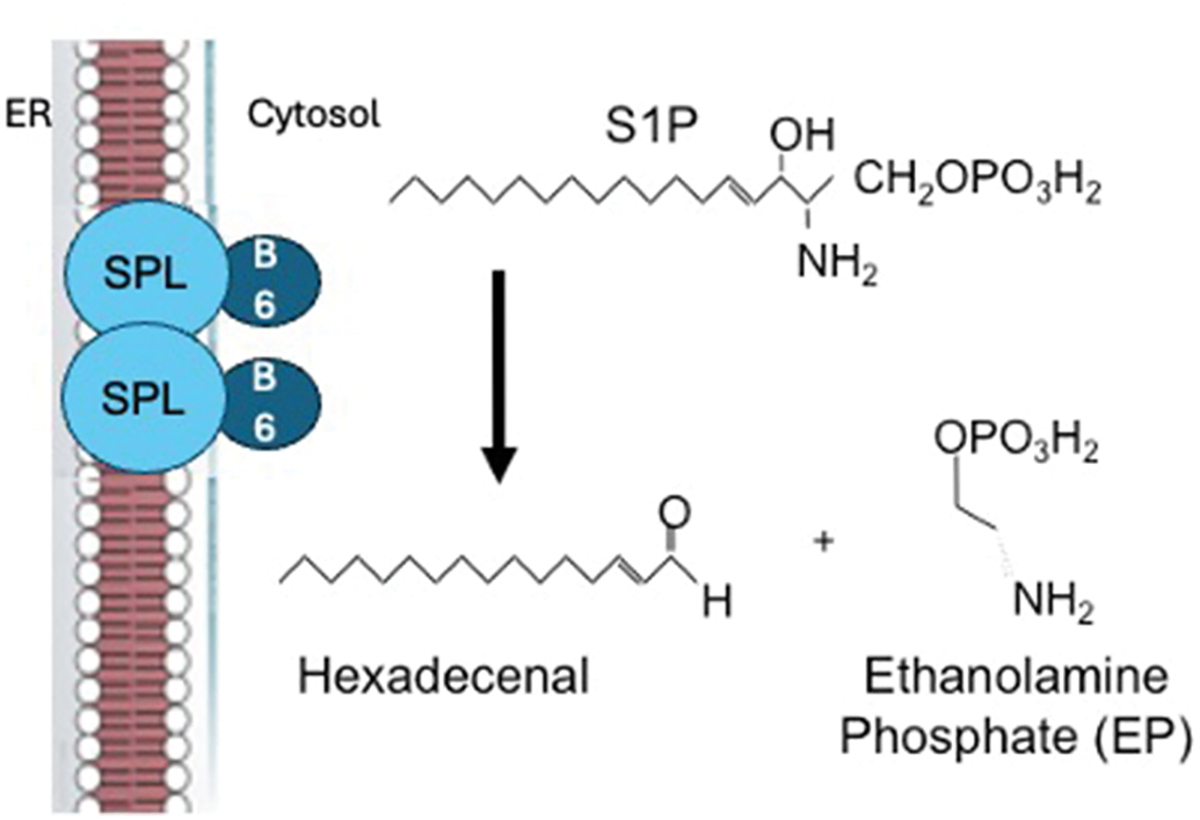
Sphingosine-1-phosphate (S1P) cleavage by sphingosine phosphate lyase (SPL).

Sphingosine phosphate lyase insufficiency syndrome (SPLIS), also known as NPHS14 or renal, endocrine, neurologic and immune (RENI) syndrome (OMIM # 617575)—is a rare autosomal recessive disorder first described in 2017 (Mitroi et al., 2017). SPLIS is an inborn error of metabolism resulting from biallelic mutations in the *SGPL1* gene. *SGPL1* encodes sphingosine phosphate lyase (SPL), a pyridoxal 5′-phosphate dependent enzyme located in the outer leaflet of the endoplasmic reticulum (ER) that is responsible for catalyzing the irreversible cleavage of sphingosine-1-phosphate (S1P) in the final step of the sphingolipid metabolic pathway (Fig. 1) (Van Veldhoven, 2000). S1P is a bioactive sphingolipid integral to numerous physiological processes, including immune cell trafficking, vascular integrity, and cellular signaling (Saba, 2019; Kunkel et al., 2013; Garris et al., 2014). The disruption of this pathway due to SPL dysfunction has many biochemical consequences, including the accumulation of the S1P substrate, other toxic lipid intermediates, and deficiency of the S1P cleavage products ethanolamine phosphate and hexadecenal, as shown by results in many model organisms (Bektas et al., 2010; Mendel et al., 2003; Zhao et al., 2021; Weber et al., 2009; Mitroi et al., 2016, 2017). We have observed significantly increased levels of phosphorylated and unphosphorylated sphingoid bases and ceramides, glycosylated sphingolipids (GSLs), and dihydrosphingolipids in the plasma of SPLIS patients compared to age and gender-matched controls, suggesting widespread disruption of sphingolipid metabolism and homeostasis (our unpublished data). These cumulative metabolic derangements result in multisystemic manifestations affecting kidneys, skin, and the endocrine, nervous, and immune systems (Pournasiri et al., 2022). The condition is associated with an overall mortality of about 50% within the first decade. Less than 20% of children presenting with renal features of SPLIS before their first birthday survive to age five (Keller et al., 2024). End stage kidney disease (ESKD) resulting from glomerulosclerosis, as well as infections and cardiorespiratory failure appear to be the main causes of death in SPLIS (Keller et al., 2024). A better understanding of the inborn error of immunity in SPLIS could lead to improved survival of these vulnerable children. In this article, we describe the SPLIS condition, review sphingolipid metabolism, and summarize various roles sphingolipids play in immunity. We then explore SPLIS-related immunodeficiency in greater detail by analyzing data available in the published literature supplemented by medical record reviews in ten SPLIS children.

## 2. Materials and methods

### General methodology.

This narrative review was conducted by collecting data available from SPLIS case reports and review articles, and by collecting and analyzing primary data from health care providers of SPLIS patients collected with informed consent. We relied heavily on our recent retrospective analysis of 76 SPLIS cases (Keller et al., 2024). The aim of our review was to identify trends in the immune dysfunctions associated with SPLIS, including lymphopenia, hypogammaglobulinemia, and inflammatory features, their treatment, and the broader implications of SPL deficiency on immune system regulation.

### Search strategy.

A comprehensive literature search was conducted using several electronic databases, including PubMed, Web of Science, and Scopus. The search was performed up to July 2024 and employed specific keywords and phrases such as “Sphingosine-1-phosphate lyase insufficiency syndrome,” “SPLIS,” “*SGPL1* deficiency,” “immune dysregulation,” “lymphopenia,” “hypogammaglobulinemia,” and “immune function in SPLIS.” Boolean operators were used to refine the search, ensuring the retrieval of studies that specifically addressed the immunological aspects of SPLIS. The search strategy also involved screening the reference lists of relevant articles to identify additional studies that were not captured in the initial database search.

### Inclusion and exclusion criteria.

Inclusion criteria for reported case literature were: peer-reviewed articles published in English; studies focusing on human subjects or relevant animal models that include any aspect of the immunological and hematological features of SPLIS; research articles, case studies, and reviews that discuss immune system abnormalities such as lymphopenia, hypogammaglobulinemia, and related immune dysfunctions in SPLIS; studies providing insights into the molecular and cellular mechanisms underlying immune defects in SPLIS. Exclusion criteria were: articles not published in English; conference abstracts, opinion pieces, and editorials that do not provide detailed, empirical data or comprehensive reviews. For analysis of primary clinical immunology data in ten SPLIS patients, all patients were genetically confirmed to have bi-allelic SGPL1 mutations by a commercial gene diagnostic laboratory.

### Data extraction and quality assessment.

Data extraction was carried out by two independent reviewers who identified and extracted key information from the selected studies. The extracted data included study design, patient demographics, findings related to immune function (e.g., lymphocyte counts, immunoglobulin levels), the impact of *SGPL1* mutations on immune responses, and any reported outcomes of immunological interventions. The quality of the included studies was not quantitatively assessed, as this review is narrative in nature. However, emphasis was placed on selecting studies that provided robust, detailed descriptions of their methodologies and results, particularly those that explored the immunological dimensions of SPLIS. The narrative review synthesizes these findings, drawing attention to consistent patterns and notable variations across different studies.

### Sex and gender as a variable.

Due to the rarity of the condition, limited number of known subjects, and lack of evidence for gender specific differences in SPLIS immune phenotypes, data from males and females with SPLIS were not treated or analyzed separately in this study.

### Human subjects and ethical considerations.

This study was conducted in accordance with the World Medical Association Declaration of Helsinki: Ethical principles for medical research involving human subjects. The University of California San Francisco Institutional Review Board (IRB) approved the study utilized to collect and analyze primary medical information from the medical records of SPLIS patients (Protocol 20–32291; activation 08–27-2024; expiration 03–24-2027). Written informed consent for the acquisition of genetic, molecular, and clinical data was obtained from the enrolled patients or from the parents of the enrolled patients except in cases wherein the information was published and only data from published reports were used for information collection. The review process adhered to ethical standards for reporting and data management, ensuring that all findings are accurately and responsibly represented and shared publicly in de-identified format. The review was conducted following guidelines for narrative reviews, with a commitment to transparency and rigor in synthesizing the literature.

## 3. The intersection of SPLIS, sphingolipids, cell signaling, and immunity

### 3.1. Clinical features of SPLIS

SPLIS presents as a multisystem disorder with a broad spectrum of clinical features. Aside from the immunological defects, the main features involve the kidney, endocrine system, central and peripheral nervous system, and skin.

Nephrotic syndrome: Nephrotic syndrome, a condition in which the kidney inappropriately spills high molecular weight proteins into the urine, is a hallmark of SPLIS. In SPLIS, this is a monogenic, steroid-resistant form and is often associated with focal segmental glomerulosclerosis pathology and podocyte foot process effacement. Patients frequently progress to ESKD within ten years of the onset of proteinuria, requiring dialysis or kidney transplantation (Keller et al., 2024). Early onset of nephrotic syndrome is a major determinant of prognosis in SPLIS, with significantly higher mortality observed in early-onset cases (Keller et al., 2024). While the underlying pathomechanism of SPLIS nephropathy remains incompletely understood, a large body of evidence supports the role of S1P signaling in fibrosis of the lung, liver, heart and other tissues (Zhang et al., 2018; Zhao et al., 2018; Shea and Tager, 2012; Huwiler and Pfeilschifter, 2018; Wang et al., 2018). Further, *SGPL1* itself has been shown to exert a protective role against fibrosis. The S1P/SPL axis acts at least in part through activation of TGFβ/SMAD signaling and extracellular matrix remodeling [ (Bhattacharyya et al., 2024) (Huang et al., 2015)].

Endocrine defects: Primary adrenal insufficiency is another hallmark of SPLIS, presenting with features such as hyperpigmentation, hypotension, and electrolyte imbalances. This condition results from impaired steroidogenesis due to adrenal gland dysfunction [ (Zhao et al., 2021) (Maharaj et al., 2022) (Maharaj et al., 2020a)]. Glucocorticoid hormone (cortisol) deficiency is commonly observed, with some patients manifesting mineralocorticoid (aldosterone) and sex hormone (testosterone) deficiencies. In boys, this can result in gonadal dysgenesis. Hypothyroidism may also be present in SPLIS. Hormone replacement is critical for managing this condition and preventing life-threatening adrenal crises (Saba, 2019). There is evidence for a mitochondrial defect that may underlie the endocrine manifestations of SPLIS (Maharaj et al., 2020a). However, adrenal calcifications are observed in many SPLIS patients, a phenotype shared with other lipid storage disorders. This suggests that lipid peroxidation could be an additional cause of adrenal gland damage.

Neurological features: Neurological symptoms include peripheral neuropathy, motor deficits, and, in some cases, developmental delay or intellectual disability. Magnetic resonance imaging of the brain may reveal abnormalities in the basal ganglia, reflecting the impact of sphingolipid accumulation on the nervous system (Martin et al., 2020). These neurological symptoms contribute significantly to the morbidity associated with SPLIS. Axonal degeneration has been observed in the peripheral nerves of SPLIS patients (Atkinson et al., 2017). Data from mouse models of SPL insufficiency in which *Sgpl1* is disrupted in a brain-restricted manner demonstrate defects of proteostasis, autophagy, disrupted metabolism of sphingolipids to phospholipids, and the accumulation of aggregation-prone proteins such as Tau, all of which may contribute to neurological dysfunction in SPLIS [ (Mitroi et al., 2016) (Mitroi et al., 2017) (Alam et al., 2020; Hagen-Euteneuer et al., 2020; Karaca et al., 2014)].

Cutaneous and systemic features: Skin manifestations (ichthyosis and acanthosis) are common in SPLIS. These features may be due to dysregulated metabolism of long chain aldehydes and specialized ceramides known to be important for skin barrier function [ (Holleran et al., 2006) (Nakahara et al., 2012)]. Other systemic features that have been reported in SPLIS include cardiomyopathy, retinopathy, failure to thrive, food intolerance, and growth failure (Keller et al., 2024). Less is known about the etiology of these phenotypes.

Immunological features: SPLIS patients may exhibit lymphopenia. This reduction in lymphocyte counts severely compromises the adaptive immune response, increasing the risk of infections. Some patients also exhibit hypogammaglobulinemia (Pournasiri et al., 2022). The precise mechanisms underlying these immune defects are thought to involve disruptions in S1P receptor signaling and impaired lymphocyte trafficking (Garris et al., 2014). Of all the SPLIS features, the pathophysiology of SPLIS-associated lymphopenia is best understood. This is because of the established role of S1P signaling in T cell egress (Matloubian et al., 2004). SPL generates the S1P gradient that T cells follow as they exit the thymus and peripheral lymphoid organs and enter the blood [ (Zamora-Pineda et al., 2016) (Schwab et al., 2005)]. Without SPL, there is no localized S1P gradient, and lymphocytes are retained in lymphoid organs, causing lymphopenia. Despite this knowledge, the detailed characterization of immunological features in SPLIS patients is lacking. In the next section, sphingolipid metabolism and the activities of selected bioactive sphingolipids are reviewed, with a focus on the influence on immunity and infectious diseases. Following that, a detailed analysis of SPLIS-related immunodeficiency is provided.

### 3.2. Sphingolipid metabolism

**Fig. 2.**
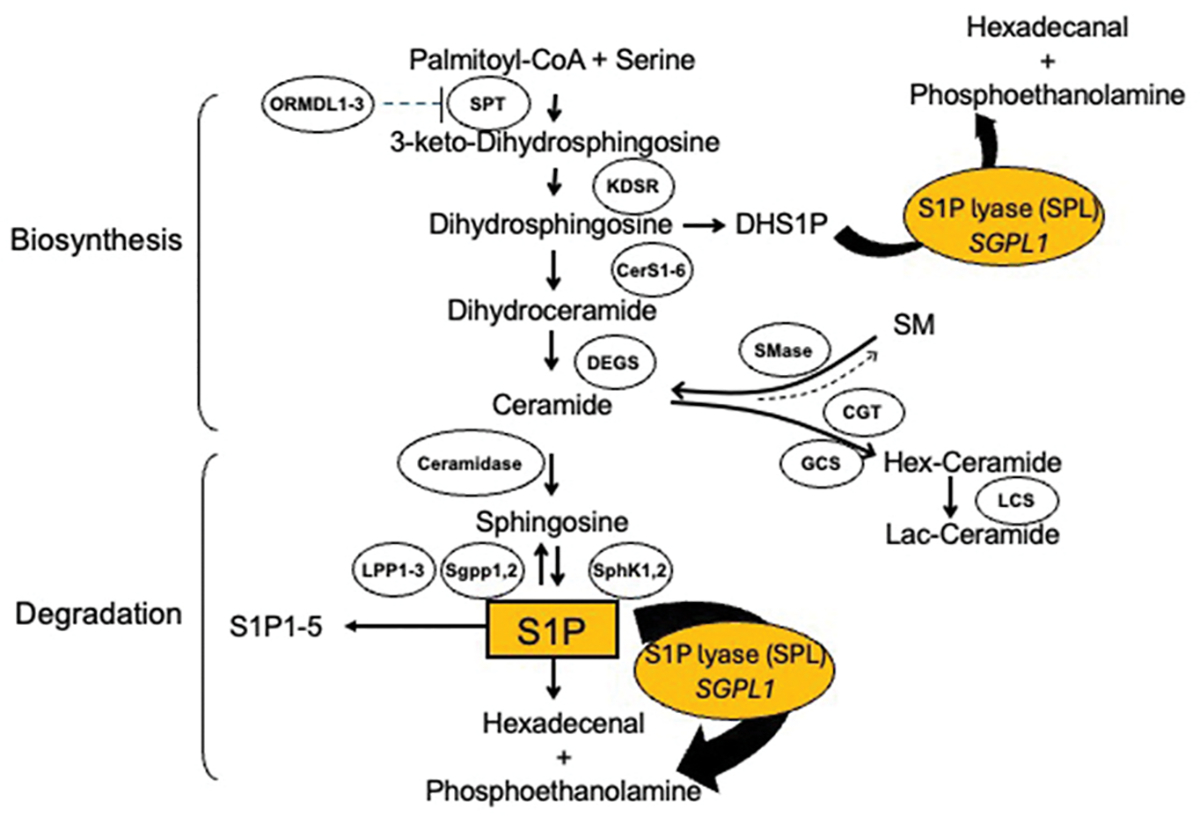
Complex pathophysiology of SPL insufficiency syndrome (SPLIS).

**Fig. 3.**
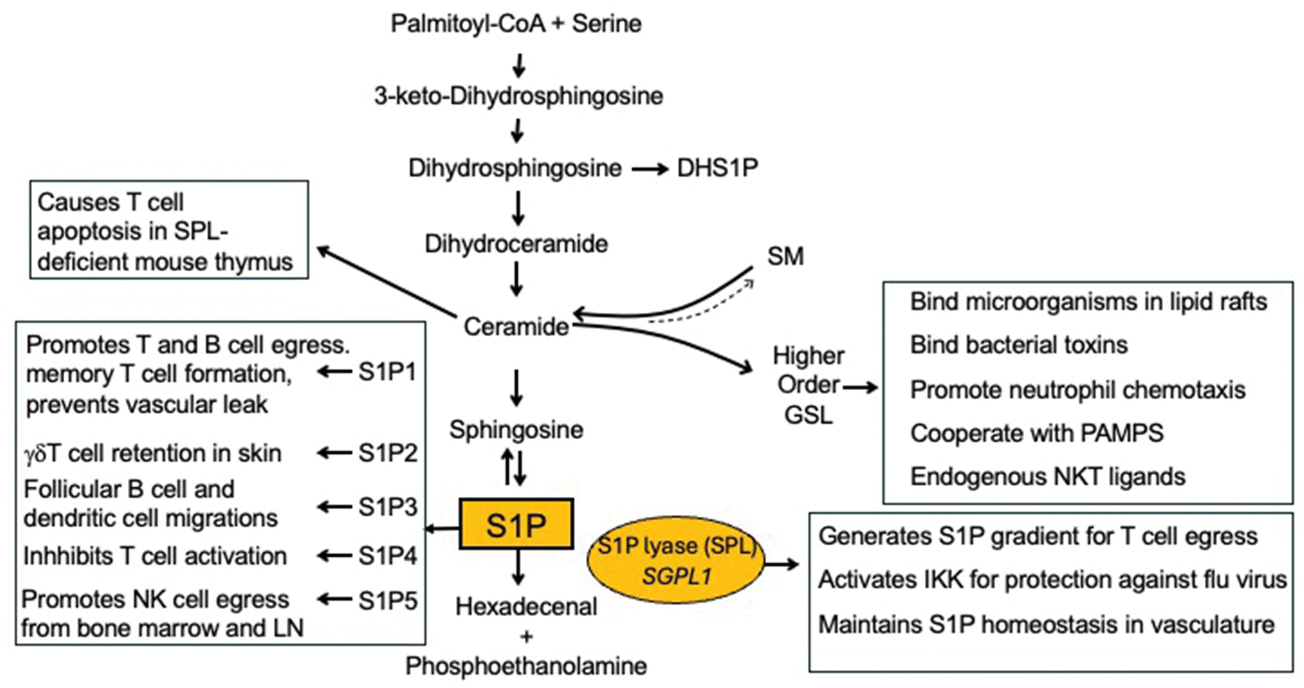
Disruption of sphingolipid metabolism in SPL insufficiency syndrome (SPLIS).

The pathophysiology of SPLIS and its many manifestations is proving to be as complex as the sphingolipid biochemical pathway it regulates (Fig. 2). Sphingolipids are vital components of cell membranes and perform important functions in cellular signaling and human physiology. Sphingolipid metabolism generates a wide range of sphingolipid species including ceramides, sphingomyelin, GSLs, S1P, and other related molecules. Importantly, these sphingolipids serve as structural components of cell membranes and act as intracellular and extracellular signaling molecules, influencing cell survival, differentiation, and apoptosis (Gault et al., 2010). Genetic mutations, such as those affecting the *SGPL1* gene in SPLIS, cause the accumulation or depletion of specific sphingolipid metabolites, leading to disturbances in cellular homeostasis and the development of certain clinical disorders (Dunn et al., 2019). In SPLIS, the only exit point of sphingolipid metabolism is blocked, resulting in a more profound disruption of the pathway than perhaps any other sphingolipid disorder (see Fig. 3).

#### Sphingolipid biosynthesis

3-Keto dihydrosphingosine, the first intermediate of sphingolipid biosynthesis, is formed by the condensation of serine and palmitoyl-CoA (Fig. 2). This initial and rate-limiting step in the *de novo* pathway is catalyzed by serine palmitoyltransferase (SPT), located in the ER (Gault et al., 2010). SPT requires a pyridoxal 5′-phosphate cofactor. This intermediate is then reduced by 3-ketodihydrosphinganine reductase (KDSR) to form dihydrosphingosine, which serves as the substrate for ceramide synthase, which in mammalian cells is represented by six isoforms (Cer1–6). Ceramide synthase acylates dihydrosphingosine with a fatty acid molecule to produce dihydroceramide. The myriad fatty acids varying by chain length and saturation that can be added to the sphingoid base at this step result in approximately two hundred different forms of dihydroceramide, which are then desaturated by dihydroceramide desaturase (DEGS) to form ceramide, the central metabolite in sphingolipid metabolism. Ceramides provide negative feedback regulation through ORMDL proteins to inhibit SPT (Breslow et al., 2010).

Ceramide is a precursor for more complex sphingolipids, including sphingomyelin and GSLs. For instance, sphingomyelin is synthesized by sphingomyelin synthase, which transfers a phosphocholine group from phosphatidylcholine to ceramide. GSLs are formed by adding sugar moieties to ceramide by glycosyltransferases in the Golgi apparatus. Specifically, addition of a glucose at the C1 position of ceramide by glucosylceramide synthase (GCS) or addition of a galactose at the same site by ceramide galactosyltransferase (CGT) produce the hexosylceramides. Glucosylceramide can be further modified to lactosylceramide by lactosylceramide synthase (LCS). These are further modified by a panoply of enzymes, resulting in higher order sphingolipids. Complex sphingolipids are integral to cell membrane structure and function, signal transduction, cell-cell interactions, myelin formation, and the formation and specific characteristics of lipid subdomains or rafts (Iwabuchi, 2018).

#### Sphingolipid degradation

The breakdown of higher order sphingolipids occurs primarily in the lysosome, where complex sphingolipids are degraded into their constituent components by enzymes with optimal activity at low pH. For instance, sphingomyelin is hydrolyzed by acid sphingomyelinase (ASM) to generate ceramide, while GSLs are broken down by various glycosidases. The regulation of higher order sphingolipid degradation is critical for maintaining cellular homeostasis. Dysregulation of this process, as seen in lysosomal storage disorders (e.g., Niemann-Pick disease, Gaucher’s disease), leads to the accumulation of sphingolipid intermediates, resulting in cellular dysfunction and disease. Many of these lysosomal sphingolipid degradative enzymes have non-lysosomal isoforms that function either at neutral or alkaline pH and are involved in cell signaling. A growing list of atypical sphingolipid disorders associated with defects in non-lysosomal sphingolipid degradative enzymes is emerging with the advent of diagnostic next generation DNA sequencing (Dunn et al., 2019). SPLIS falls into this category of recently recognized conditions. Such conditions are revealing the important roles of sphingolipids in other aspects of physiology.

The common degradative pathway begins with the activity of ceramidases which can cleave ceramide into sphingosine, followed by phosphorylation of sphingosine to S1P by sphingosine kinase 1 or 2 (SphK1,2). S1P can be dephosphorylated intracellularly by S1P phosphatases (Sgpp1,2) or extracellularly by lipid phosphate phosphatases (LPP1–3). This route of S1P metabolism regenerates sphingosine, which can be re-phosphorylated by the sphingosine kinases, creating a useful mechanism for controlling S1P-mediated signals, but also resulting in an endless metabolic cycle. Alternatively, S1P can be degraded by S1P lyase (SPL), which irreversibly cleaves S1P at the C2–3 carbon-carbon bond (Fig. 1). Note that SPL can also degrade dihydrosphingosine-1-phosphate (DHS1P) generated by SphK1,2 during *de novo* synthesis, although the cellular and circulating content of DHS1P is much less than that of S1P. The critical role of SPL is due to the irreversibility of its actions, such that it controls the levels of S1P in cells, tissues and blood/lymph. In SPLIS, mutations in the *SGPL1* gene result in the dysfunction of SPL due to reduced catalysis and protein destabilization causing reduced SPL protein abundance. Ultimately, SPL insufficiency leads to the accumulation of S1P and disruption of the delicate balance of sphingolipids in cells [ (Bektas et al., 2010) (Taylor et al., 2019)]. This imbalance plays a significant role in the pathogenesis of SPLIS, contributing to the various clinical symptoms observed in affected individuals. Like SPT, the first enzyme of the *de novo* pathway, this last enzyme in the pathway requires a pyridoxal 5′-phosphate cofactor to mediate its catalytic function.

### 3.3. The role of sphingolipids in cell signaling

S1P is a potent signaling molecule that plays crucial roles in immune cell trafficking, inflammation, fibrosis, vascular development, and cell survival. S1P signals through a family of five G-protein-coupled receptors (S1P1-S1P5), each having distinct functions depending on the tissue and cell type in which it is expressed [ (Garris et al., 2014)(Graler, 2010)]. One of the most clinically important functions of S1P signaling is its ability to regulate lymphocyte egress from lymphoid organs into the bloodstream, a process essential for maintaining immune surveillance. The role of S1P and other sphingolipids in immunity will be addressed below.

S1P is a crucial mediator of vascular homeostasis. S1P signaling via S1P1 and S1P3 receptors on endothelial cells promotes endothelial barrier integrity, preventing vascular leakage and maintaining normal blood vessel function (Baeyens and Schwab, 2020). S1P stimulates the migration, proliferation, and survival of endothelial cells, which is essential for maintaining vascular stability and promoting angiogenesis [ (Paik et al., 2004) (Kono et al., 2004)]. In SPLIS patients, the dysregulation of S1P levels due to impaired degradation likely affects vascular integrity. The excess S1P leads to aberrant endothelial cell signaling, which can result in increased vascular permeability and edema. SPLIS patients already suffer edema associated with loss of albumin and low oncotic pressure in the blood due to nephrotic syndrome. In these patients, the loss of vascular barrier function would be expected to cause further pulmonary and soft tissue edema. Additionally, S1P plays a key role in the regulation of the blood-brain barrier (Noguchi and Chun, 2011). It regulates calcium homeostasis and has neurotoxic properties [ (Hagen et al., 2011) (Hagen et al., 2009)]. Its dysregulation may contribute to the neurological complications observed in SPLIS patients, such as developmental delays and seizures.

Ceramide, a key intermediate in sphingolipid metabolism, is a bioactive lipid that regulates cellular stress responses, including apoptosis, senescence, and autophagy. It is produced not only via the *de novo* pathway but also through the hydrolysis of sphingomyelin by sphingomyelinases, which generate ceramide in response to extracellular stress signals such as inflammatory cytokines, chemotherapy, or oxidative stress (Won and Singh, 2006). Ceramide acts as a second messenger in signaling pathways that lead to programmed cell death, particularly by activating protein phosphatase 2A and other phosphatases, which dephosphorylate and inactivate pro-survival signaling proteins such as AKT and ERK (Gault et al., 2010). This deactivation promotes apoptosis, making ceramide a central mediator of cell death in response to stress (Cuvillier et al., 1996). This activity may aggravate SPLIS lymphopenia, as murine studies have shown that ceramide accumulates in *Sgpl1* knockout mouse thymii, causing T cell apoptosis (Weber et al., 2009). Ceramide also forms ceramide-enriched platforms on the plasma membrane, which facilitate the clustering of signaling molecules and receptors, such as death receptors (e.g., Fas, TNF-R), that initiate apoptosis (Grassme et al., 2001). These platforms provide a structural framework for the initiation and amplification of apoptotic signals. In addition, higher order sphingolipids may act as microbial receptors and may further shape the immune response to pathogens through interaction with sensors and effectors of the innate and adaptive immune response.

Sphingolipids, particularly sphingomyelin, are abundant in the central nervous system (CNS), where they are major components of neuronal membranes and myelin sheaths. Sphingomyelin is critical for maintaining the structural integrity of myelin, which insulates axons and facilitates the rapid transmission of electrical signals between neurons (McCluskey et al., 2022). In SPLIS, the disruption of sphingolipid metabolism affects the CNS, contributing to neurological symptoms such as peripheral neuropathy, developmental delays, and intellectual disability. The accumulation of S1P and other sphingolipid metabolites in neural tissues disrupts neuronal function and can lead to neurodegeneration. Additionally, the role of S1P in regulating the blood-brain barrier may explain the neurological manifestations in SPLIS patients [ (Martin et al., 2020) (Alam et al., 2020)]. Beyond its structural role, S1P also influences neural development and neuroinflammation. S1P signaling through S1P1 has been shown to regulate the migration of neural progenitor cells and modulate inflammatory responses in the brain (Callihan and Hooks, 2012). In conditions such as multiple sclerosis, where neuroinflammation is a key feature, the S1P receptor modulator fingolimod has been used to reduce the migration of autoreactive lymphocytes into the CNS and to diminish neuroinflammation, thereby mitigating disease progression (Kunkel et al., 2013).

Sphingolipids, particularly ceramide and S1P, play important roles in maintaining lung structure and function. Ceramide accumulation in lung tissues has been implicated in the pathogenesis of chronic lung diseases such as chronic obstructive pulmonary disease and idiopathic pulmonary fibrosis. Ceramide promotes apoptosis of alveolar epithelial cells and fibroblasts, contributing to lung tissue damage and fibrosis (Ziobro et al., 2013). In contrast, S1P plays a role in regulating airway inflammation and bronchoconstriction in diseases such as asthma. Elevated levels of S1P in the airways can lead to enhanced bronchoconstriction and airway hyperresponsiveness, exacerbating the symptoms of asthma. S1P signaling also influences the migration of immune cells into the lungs during inflammatory responses, further contributing to the pathophysiology of respiratory diseases.

Sphingolipids are increasingly recognized for their roles in metabolic regulation, particularly in the context of obesity, insulin resistance, and type 2 diabetes. Ceramide interferes with insulin signaling by inhibiting the activation of Akt, a key kinase in the insulin signaling pathway. This results in reduced glucose uptake by adipocytes and skeletal muscle cells, contributing to insulin resistance (Bikman and Summers, 2011). In SPLIS, the accumulation of sphingolipid metabolites may exacerbate metabolic dysfunction. For example, elevated ceramide levels in adipose tissue and skeletal muscle impair insulin sensitivity, leading to metabolic disturbances. Additionally, sphingolipids play a role in regulating lipid metabolism in the liver, and their dysregulation can contribute to conditions such as non-alcoholic fatty liver disease[(Wang et al., 2022) (Roszczyc-Owsiejczuk and Zabielski, 2021)].

### 3.4. Sphingolipids in immunity

Sphingolipids are involved in signaling pathways that regulate both innate and adaptive immune responses. They are crucial in modulating immune cell trafficking, cytokine production, and apoptosis, as well as controlling the inflammatory responses that protect the body from infections and tissue damage. They also play roles in pathogen recognition and innate immune responses. The most well-known sphingolipids involved in these processes include ceramide and S1P.

#### S1P as a central mediator of adaptive immunity

S1P is a critical factor in the adaptive immune response. As T cells mature in the thymus, they increasingly express more S1P1 on their cell surface, with a 3-log increase from double negative to single positive cells at the final stages of T cell maturation (Baeyens and Schwab, 2020). The activation of S1P1 on the surface of these mature T lymphocytes guides them along an S1P gradient that is low in lymphoid tissues and high in the circulation. This gradient, generated by SPL activity in dendritic cells located at the corticomedullary junction of the thymus, with additional help from lipid phosphatases, is responsible for guiding lymphocytes out of the thymus, allowing them to patrol the body for potential pathogens (Zamora-Pineda et al., 2016). A similar mechanism guides activated T cells to egress from peripheral lymphoid organs to enter the lymphatics and, eventually, the blood. In SPLIS patients, mutations in the *SGPL1* gene result in defective S1P degradation, causing its accumulation and disrupting the S1P gradient in the thymus, peripheral lymphoid organs and other tissues. This leads to impaired lymphocyte egress, contributing to lymphopenia and immune system dysfunction (Schwab and Cyster, 2007).

In addition to controlling mature T cell egress, S1P concentration gradients between tissues and the circulatory system guide the migration of immune cells such as T cells, B cells, natural killer (NK) cells, plasma cells, hematopoietic stem cells, monocytes/macrophages, and dendritic cells (Baeyens and Schwab, 2020). S1P1 is the key receptor that mediates the exit of lymphocytes from the thymus and secondary lymphoid organs, allowing them to enter circulation and carry out immune surveillance (Matloubian et al., 2004). S1P1 also promotes naïve T cell survival and is required for T cells to form memory T cells after exit from lymph nodes after exposure to antigen (Mendoza et al., 2017). S1P1 may also contribute to efficient T and B cell egress across the endothelial barrier (Nakai et al., 2014; Benechet et al., 2016; Grigorova et al., 2009; Pham et al., 2008), and egress of immature B cells from the bone marrow (Pereira et al., 2010). S1P1 drives follicular B cell exit from the spleen into the general circulation (Arnon et al., 2013). It enables other immune cell types including plasma cells and hematopoietic stem cells to exit the spleen, and it influences the migration of marginal zone B cells within different anatomical regions of the spleen (Arnon et al., 2013).

Other S1P receptors play key roles in other aspects of immunity (Baeyens and Schwab, 2020). For example, S1P1 and S1P3 are both needed for dendritic cell egress from tissues into the lymphatics (Lamana et al., 2011; Pham et al., 2010; Rathinasamy et al., 2010). S1P3 supports follicular B cell chemotaxis within the spleen [ (Cinamon et al., 2004) (Sinha et al., 2009)]. S1P2 signaling is needed to confine B and T cells to splenic locations [ (Green et al., 2011) (Moriyama et al., 2014)], and it helps retain γδT cells in inflamed skin (Laidlaw et al., 2019). S1P5 on NK cells is necessary for their egress from bone marrow and lymph nodes, and for their strategic positioning and entry into the lymphatics [ (Jenne et al., 2009) (Walzer et al., 2007)]. S1P2 modulates immune cell adhesion and migration, particularly in vascular inflammation (Zhang et al., 2013), while S1P3 influences the recruitment of immune cells to inflammatory sites [ (Niessen et al., 2008) (Murakami et al., 2014)]. S1P3 appears to be important in the regulation of neuroinflammation, itch and pain (Hill et al., 2018a, 2018b; Camprubi-Robles et al., 2013). The diversity of S1P receptors enables fine-tuned control over immune cell behavior in various contexts. All signaling events mediated through the S1P receptors may become up- or downregulated by high S1P levels and aberrant signaling or receptor desensitization in SPLIS patients, thereby contributing to immune dysregulation and ineffectual response to pathogens.

#### Ceramide’s role in innate immune function

Ceramide is another sphingolipid with critical roles in immune regulation, particularly in mediating apoptosis and inflammatory responses (Cuvillier et al., 1996). Ceramide promotes apoptosis, a process essential for eliminating damaged or infected cells during immune responses (Albeituni and Stiban, 2019). Ceramide also regulates inflammatory pathways by forming ceramide-enriched lipid rafts in the plasma membrane. These lipid rafts act as platforms for receptor clustering and activation, particularly in pro-inflammatory signaling pathways. The ceramide-induced activation of the nuclear factor kappa-light-chain-enhancer of activated B cells (NFκB) pathway leads to the production of pro-inflammatory cytokines such as tumor necrosis factor-alpha (TNFα) and interleukin-6 (IL-6) (Albeituni and Stiban, 2019). These cytokines are critical in orchestrating immune responses to infections and tissue injury. Ceramide also modulates macrophage activity, enhancing their ability to produce cytokines and carry out phagocytosis. The accumulation of ceramide in macrophages is essential for the clearance of pathogens and apoptotic cells, highlighting its importance in maintaining immune homeostasis. Because ceramides have been found to be elevated in animal models of SPLIS, it is possible that hyperactivation of pro-inflammatory pathways such as TNFα, NFκB, and IL-6 play a role in causing SPLIS organ damage and pathology (Albeituni and Stiban, 2019).

Ceramide also plays a key role in the host defense against bacterial infections by regulating autophagy, a process in which cells degrade and recycle damaged components and pathogens. Ceramide-mediated autophagy is particularly important in the clearance of intracellular pathogens such as *Mycobacterium tuberculosis*. By enhancing ceramide levels, the immune system can more effectively eliminate bacterial infections and reduce infection-related inflammation (Grassme and Becker, 2013).

#### Glycosylated sphingolipids in immune cell activation

GSLs are a subclass of sphingolipids that play significant roles in both the innate and adaptive immune responses (Mauri et al., 2018). These lipids are localized on the outer leaflet of the plasma membrane, where they participate in cell-cell interactions, antigen presentation, and immune cell activation. GSLs are crucial components of lipid rafts, which organize signaling molecules and receptors necessary for the activation of immune cells such as macrophages, neutrophils, and T lymphocytes. In the innate immune system, GSLs function as pattern recognition receptors (PRRs), recognizing pathogen-associated molecular patterns (PAMPs) on microbial surfaces (Iwabuchi, 2018). For example, bacterial lipopolysaccharides (LPS) bind to GSL-enriched lipid rafts, facilitating pathogen recognition by macrophages and dendritic cells. This interaction triggers downstream signaling pathways that lead to the production of pro-inflammatory cytokines and the recruitment of immune cells to the site of infection [ (Nakayama et al., 2013) (Zhang et al., 2019)]. Lactosylceramide (LacCer), a specific GSL, plays a key role in neutrophil activation and phagocytosis. LacCer enhances the ability of neutrophils to engulf and destroy microbial pathogens, contributing to effective host defense. GSLs also regulate adaptive immune responses by organizing major histocompatibility complex molecules and facilitating antigen presentation to T cells (Iwabuchi et al., 2015) (Iwabuchi et al., 2022)]. We have observed shunting of ceramides into the GSL pathway in SPLIS patients, based on the finding of increased levels of plasma hexosylceramides (our unpublished data). Thus, disturbances in raft composition and other innate immune functions of GSLs may conceivably play a role in SPLIS immunodeficiency states.

#### Sphingolipids in viral infections

S1P signaling has been implicated in controlling immune cell responses to viral infections, such as influenza and HIV. In influenza infection, S1P receptor signaling through S1P1 limits excessive lung inflammation, reducing the severity of infection-related cytokine amplification (Teijaro et al., 2011). S1P signaling is involved in regulating the migration of immune cells to sites of infection, as well as controlling the production of cytokines and interferons that are essential for antiviral defense. In the case of influenza, S1P signaling through S1P1 limits excessive lung inflammation during infection, preventing tissue damage while allowing the immune system to clear the virus. However, excessive S1P signaling can lead to immune suppression and allow the virus to evade the immune response. Therapeutic modulation of S1P signaling during viral infections is being explored as a means to balance immune activation and resolution, reducing tissue damage while promoting viral clearance. Ceramide is involved in the fusion of the HIV virus with host cell membranes, facilitating viral entry and replication (Barklis et al., 2021; Finnegan et al., 2004; Lund et al., 2006). Inhibiting ceramide production in HIV-infected cells has been shown to reduce viral replication and improve the effectiveness of antiretroviral therapies. Additionally, S1P receptor modulators are being studied for their potential to enhance immune responses in individuals with HIV by promoting the migration of immune cells to sites of viral infection [ (Avota et al., 2021) (Thomas et al., 2023)].

Importantly, SPL has an antiviral function against influenza A virus which is mediated by its by interacting with IKKε, thereby promoting the type I interferon (IFN) innate immune response to infection with the virus [ (Vijayan et al., 2017) (Wolf et al., 2021)]. The NS1 protein of influenza A virus hampers this by triggering proteasomal SPL degradation, which reduces the type I IFN innate immune response. Loss of SPL activity in response to influenza A infection in SPLIS patients could be catastrophic due to the already compromised SPL activity in these patients. There is a risk that these mechanisms are operational in other viral infections as well.

## 4. Detailed analysis of the immunological features of SPLIS

To dive deeper into the immunological features of SPLIS, we performed a systematic search of databases to collect clinical information on the immunological and infectious disease manifestations of SPLIS. We included data from case reports of SPLIS patients from 2017, when the initial genetic description of the syndrome was published, to the present [(Lovric et al., 2017) (Prasad et al., 2017) (Pournasiri et al., 2022) (Keller et al., 2024) (Atkinson et al., 2017) (Taylor et al., 2019) (Maharaj et al., 2020b; Janecke et al., 2017; Linhares et al., 2017; Pezzuti et al., 2014; Settas et al., 2018; Tran et al., 2022; Yang et al., 2023; Roa-Bautista et al., 2023; Spizzirri et al., 2023; Tastemel et al., 2023; Seven Menevse et al., 2022; Najafi et al., 2022; Ron et al., 2022; Zhao et al., 2020; Bamborschke et al., 2018; Saygili et al., 2019; Mathew et al., 2022; Buyukyilmaz et al., 2023)]. We relied heavily on results summarized in a retrospective analysis of 76 SPLIS patients reported by our group in 2024 (Keller et al., 2024). An additional two SPLIS patients are known to us that remain unreported. In addition, detailed clinical immunology, hematology, and treatment data on ten SPLIS patients was collected from colleagues caring for these patients. Data from these various sources were non-uniform and included information on the following parameters (with age-dependent reference values when relevant): general description of the immune disorder, leukocyte count, absolute lymphocyte count and percentage, absolute neutrophil count and percentage, absolute monocyte count and percentile, absolute CD3^+^ (T cell), CD4^+^ T cell, CD8^+^ T cell, CD19^+^ B cell, CD16/56+ NK cell counts, Red blood cell indices, platelet count, hypogammaglobulinemia (yes/no), serum IgG, IgA, IgM levels, complement C3 and C4, T cell proliferation study results, response to childhood vaccinations, documented infections, C reactive protein, and T cell receptor excision circles (TREC). Treatment modalities surveyed included infection prophylaxis and blood product preparation.

### Lymphopenia and hypogammaglobulinemia in SPLIS

We analyzed the status of immune function in 46 evaluable SPLIS patients (Table 1). Evaluable patients were defined as patients for whom lymphopenia was noted and/or immunoglobulin levels and/or lymphocyte subset results were available in published reports or available patient records. Immunodeficiency was defined by exhibiting at least one of the three key parameters of immune dysfunction, namely lymphopenia, hypogammaglobulinemia, and/or low T and B cell subsets. Lymphopenia was observed in 35 patients. Hypogammaglobulinemia (low serum IgG, IgM and/or IgA levels for age) was observed in 20 patients. This condition is indicative of compromised humoral immunity, which is consistent with the expected immunodeficient profile. Low T, B, and NK cell subsets were identified in several patients based on reduced CD3, CD4, CD8, CD19, and CD16^+^CD56 counts compared to normal ranges. In total, 20 patients exhibited deficiencies in these critical immune cell populations. By evaluating these parameters collectively, 43 of 46 evaluable patients were classified as having immunodeficiency, representing 93% of the total evaluable cases of SPLIS and 55% of known SPLIS cases in total. This is particularly notable, considering that for most blood tests, “normal” is within a 95% confidence level, meaning that approximately 5% of the population is expected to be outside the normal range, compared to the much higher percentages found in the SPLIS population.

Table 1 summarizes the findings regarding T cell proliferative responses, responses to childhood vaccinations, and TREC values for the patients in our study. T cell proliferative responses to mitogens (PHA, Con A, and PWM) were abnormal in 40% of SPLIS patients, but the number of patients with detailed T cell function assays were limited. Regarding childhood vaccinations, a range of responses to anti-hepatitis B (anti-HBs), tetanus, diphtheria, pneumococcal, and MMR (measles, mumps, rubella) were observed, with most patients demonstrating protective levels. However, two patients exhibited suboptimal responses, as indicated by low antibody titers to specific vaccines. Several patients T cell receptor excision circle (TREC) testing is a newborn screen that detects recent thymic T cell emigrants based on a PCR test for T cell receptor rearrangement. TREC values were very low or absent in 6 of 7 patients in which the data was available, indicating reduction in thymic output, i.e., reduced numbers of circulating recent thymic emigrants (naïve T cells).

### Interventions and infection profile in SPLIS patients

Table 2 summarizes the types of infections and pathogens isolated, and Table 3 summarizes prophylactic interventions administered to various SPLIS patients, including prophylactic antimicrobials and intravenous and subcutaneous immunoglobulin replacement therapy.

The types of infections observed included severe systemic infections such as bacteremia, sepsis, and septic shock, as well as opportunistic infections including BK viremia (one case post kidney transplant) and Epstein Barr Virus (EBV) positivity. Recurrent infections, including oral candidiasis, lower respiratory tract infections, and catheter-related infections, were also documented. Prophylactic antimicrobials were used in various combinations, including trimethoprim/sulfamethoxazole (TMP/SMX), acyclovir, azithromycin, and fluconazole, with some patients requiring protective isolation or additional medications for *Pneumocystis jirovecii* pneumonia (PJP) prophylaxis. Immunoglobulin therapy was administered in the form of both subcutaneous (SCIg) and intravenous (IVIg) immunoglobulins. The frequency of blood products varied among patients, with some requiring frequent transfusions in early childhood and others only needing occasional or no routine blood product transfusions. When given, the products were generally irradiated and leukodepleted, but these steps were not taken if the patient’s immunity was considered uncompromised.

In summary, the immunological features of SPLIS are wide-ranging. Most commonly, SPLIS patients exhibit T cell-specific lymphopenia, consistent with the established role of the S1P/S1P1/SPL axis in lymphocyte egress. However, hypogammaglobulinemia, diminished responses to childhood vaccinations, and opportunistic infections with bacterial, viral and fungal pathogens were observed. TREC testing has identified some patients in the newborn period. Lymphopenia has been reported to improve after cofactor supplementation in some SPLIS patients, indicating upregulation of SPL activity (Zhao et al., 2020). Major findings may be attributed to aberrant S1P signaling and S1P receptor desensitization leading to a block in lymphocyte egress in SPLIS patients. However, immunosuppressive regimens post-kidney transplant may create an even more immunologically vulnerable state in these patients. Further study will be required to fully elucidate the complex mechanisms underlying SPLIS immunodeficiency and determine the most effective prophylaxis against infection.

## 5. Discussion

Sphingolipids are essential regulators of immune cell function, inflammation, and apoptosis, making them critical players in a wide range of diseases, including autoimmune diseases, cancer, neurodegenerative diseases, and metabolic disorders. The roles of sphingolipids in immune cell trafficking, cytokine production, fibrosis, and cell death underscore their importance in both innate and adaptive immunity. A wide range of sphingolipids contribute to immune function, with S1P being most well-recognized and leveraged (by the pharmacological targeting of S1P1) for treating autoimmune diseases. However, S1P has broader impact on innate and adaptive immunity; other sphingolipids, including ceramides and GSLs, play additional supportive roles.

SPLIS is a complex and challenging disorder that causes a global disruption of sphingolipid metabolism with broad biochemical consequences. It is characterized by fibrotic kidney disease leading to ESKD and a requirement for renal replacement therapy. This feature has perhaps overshadowed the other SPLIS manifestations, including the recognition of this condition as a primary immunodeficiency. In fact, our retrospective analysis of SPLIS natural history in 76 patients revealed infection/sepsis as the second leading cause of death in children with SPLIS. This suggested to us that a deeper investigation of the immunological features of SPLIS as well as the clinical practices of prophylaxis and treatment of SPLIS patients was warranted.

We observed a wide range of immune function in the 46 patients for whom sufficient data were available for analysis, out of a total of 78 known SPLIS patients. Immunodeficiency—as defined by either lymphopenia on complete blood count, low T and/or B cell subsets, and/or hypogammaglobulinemia— was found in more than half of SPLIS cases. This is likely an underestimate, considering the number of patients lacking clinical immunology data. Low absolute lymphocyte counts were the most common finding. A low absolute lymphocyte count might appear relatively unremarkable in patients with severe kidney, endocrine and neurological problems requiring emergent response. When immunology results were abnormal, T cells were predominantly affected, consistent with the essential role of S1P1 in T cell egress compared to the secondary role S1P signaling plays in B and NK cell egress. However, some cases demonstrated low levels of B and NK cell subsets as well.

In the small number of cases where functional assays of SPLIS patient T cells were tested, results were normal. In contrast, the response to childhood vaccinations was sometimes diminished, indicating compromise of the adaptive immune system. Hypogammaglobulinemia was observed in 23 patients. This feature may have its etiology in the requirement of T cell input for B cell activation and immunoglobulin production. B cells were reduced in some SPLIS patients, further exacerbating immunoglobulin production. Hypogammaglobulinemia may also be caused and/or worsened by nephrotic syndrome protein losses. Preclinical studies may be needed to distinguish the specific contribution of each of these factors independently.

Bacterial, fungal and viral infections were observed in SPLIS patients, sometimes with fatal consequences. Viral infections may be particularly dangerous in SPLIS because of the known protective role of SPL in influenza A infection and potentially other viral infections, and the targeting and inactivation of SPL by these viral defense proteins. Considering the critical role of S1P signaling in T lymphocyte trafficking, its supportive role in the trafficking and spatial organization of numerous other cells of the innate and adaptive immune system, and the additional contribution to immunity/inflammation of other sphingolipids and GSLs that accumulate in SPLIS patients, it is perhaps not surprising to find SPLIS patients at risk for many types of infections. Whether chronic and sustained lymphopenia will make SPLIS patients at greater risk for leukemia, lymphoma or other malignancies is an important unanswered question that will require long-term monitoring to determine.

TREC newborn screening test results were low or completely absent in several SPLIS infants early in life. This suggests that quantifying recent thymic emigrants, which the TREC test measures, may be a more sensitive test than absolute lymphocyte count for detecting SPLIS-related immunodeficiency. TREC tests are part of the Recommended Uniform Screening Panel in the United States and are used to identify newborns with severe combined immunodeficiency (Kwan et al., 2013). Our findings suggest that SPLIS patients could be identified by this method, providing a window of opportunity for early intervention. In some SPLIS patients, responses to supplementation with pyridoxine or pyridoxal 5′-phosphate have been observed, including an increase in absolute lymphocyte counts (Zhao et al., 2020). This observation will require further substantiation in a larger cohort of patients. However, if confirmed, the potential for vitamin supplementation and early institution of measures to protect/prophylax against infection provide a rationale for newborn screening for SPLIS.

Current therapeutic strategies in SPLIS are primarily supportive, focusing on managing symptoms rather than addressing the underlying genetic defect. However, emerging therapies such as gene therapy, enzyme replacement therapy, and small molecule inhibitors offer hope for more definitive treatments in the future. Thus far, only pyridoxine therapy has shown a positive impact on immune dysfunction in SPLIS, and this has been limited to a handful of case reports. Preclinical studies in *Sgpl1* knockout mice indicate that many phenotypes associated with SPL insufficiency are corrected by adeno-associated virus mediated gene therapy. However, the one phenotype that was not corrected using AAV9 to deliver a working copy of the *SGPL1* gene was lymphopenia (Zhao et al., 2021). Therefore, it may be necessary to improve targeting to lymphoid tissues and/or perform bone marrow transplantation to address the immunodeficiency of SPLIS genetically.

Ultimately, while significant progress has been made in understanding the immunological aspects of SPLIS, many questions remain unanswered. Continued research is needed to fully elucidate the mechanisms of immune dysregulation, develop targeted therapies, and improve the quality of life for patients with SPLIS. By addressing these challenges, the medical community can move closer to providing effective and comprehensive care for those affected by this rare and debilitating syndrome.

## 6. Conclusion

SPLIS poses a distinct and complex problem in immunology, characterized by substantial impairments in immunological function. Numerous sphingolipids that contribute to regulating and shaping innate and adaptive immune responses accumulate in SPLIS patients. Lymphopenia is a defining characteristic of SPLIS, caused by impaired S1P signaling that obstructs lymphocyte egress from lymphoid organs. This immunodeficiency makes patients vulnerable to recurrent and severe infections caused by bacterial, viral, and fungal pathogens. Furthermore, a reduction in T, B, and NK cells further undermines the immune response, which is demonstrated by hypogammaglobulinemia and inadequate responses to vaccines. Hypogammaglobulinemia may arise from or be exacerbated by nephrotic syndrome. Our findings indicate a more extensive immunological dysregulation in SPLIS patients, extending beyond lymphopenia, potentially resulting from disrupted ceramide and glycosphingolipid metabolism, which may exacerbate inflammation and apoptosis in immune cells. Interventions including immunoglobulin replacement and antibiotic prophylaxis have been instituted, but their impact is not yet known, and they are supportive rather than curative. A few SPLIS patients have exhibited enhanced lymphocyte counts after cofactor supplementation, indicating potential benefit in addressing the immunological dysfunction linked to SPLIS. However, this requires further validation in larger patient cohorts. A comprehensive understanding of the immunological foundations of SPLIS is essential for the development of targeted treatments. Newborn screening techniques, including TREC testing, can detect immunodeficiency at an early stage, facilitating timely management. Progress in gene therapy and enzyme replacement therapy present prospective approaches for addressing the genetic and metabolic origins of SPLIS. Enhancing our comprehension of the immunologic landscape of SPLIS will be crucial for improving patient outcomes, determining optimal immunosuppressive regimens used in organ transplantation and gene therapy, and reducing the dangers of severe infections and long-term immune deficiencies.

## Figures and Tables

**Table 1:** Immunological parameters in SPLIS patients.

Parameter	# Among evaluated patients	# out of range (%)	Trend

WBC Count	18	10 (55.6)	Low
Absolute Lymphocyte Count	43	35 (81.3)	Low
Absolute Neutrophil Count	11	1 (9.1)	Low
Absolute Monocyte Count	10	2 (20)	Low
RBC Mass	10	6 (60)	Low
Hgb	10	5 (50)	Low
% Hct	10	4 (40)	Low
Platelet Count	11	6 (54.5)	Low
CD3^+^	23	15 (65.2)	Low
CD4^+^	23	15 (65.2)	Low
CD8^+^	23	14 (60.9)	Low
CD19^+^	16	13 (81.2)	Low
CD16^+^CD56^+^	18	8 (44.5)	Low
IgG Level	18	10 (55.5)	Low
IgA Level	18	9 (50)	Low
IgM Level	18	8 (44.5)	Low
All immunoglobulin levels (including unspecified)	26	20 (77)	Low
Complement C3	4	1 (25)	Low
Complement C4	5	2 (40)	Low
CRP Level	9	4 (44.4)	High
T cell proliferative responses	5	2 (40)	Low
Response to childhood vaccination	12	2 (17)	Low
TREC results	8	7 (87.5)	Low/Absent

T cell proliferative responses, response to vaccinations, and TREC in SPLIS.

**Table 2:** Infections and specific pathogens in SPLIS patients.

Infections	Pathogens	N. of Patients	Comment

Sepsis/Bacteriemia/Septic Shock	*Serratia marcescens*, Coagulase-negative staphylococci	11	
Catheter-related infection	N/A	1	
Gastroenteritis/Intraabdominal infection/Peritonitis	*Clostridium difficile*, Rotavirus	3	Recurrent C. difficile infections in one patient
Respiratory tract infections	Respiratory Syncytial Virus (RSV), Enterovirus	4	Reported as the cause of death in one patient
Oral infections	*Candida parapsilosis, Candida albicans*	3	
Viremia	BK Polyomavirus, Epstein-Barr Virus (EBV), Adenovirus	3	Post-kidney transplantation infection due to BK virus infection

**Table 3:** Infection prophylaxis in SPLIS patients.

Agent	N. of Patients

TMP/SMX	4
ABx (unspecified)	1
Penicillin	1
Fluconazole	2
Immunoglobulins (IVIg, SCIg)	3
Acyclovir	1
Azithromycin	1

## Data Availability

The data that has been used is confidential.

## Abbreviations:

ASM: acid sphingomyelinase
CerS1–6: ceramide synthase 1–6
CGT: ceramide galactosyltransferase
CNS: central nerous system
DHS1P: dihydrosphingosine-1-phosphate
EBV: Epstein Barr virus
ER: endoplasmic reticulum
ESKD: end stage kidney disease
FTT: failure to thrive
GCS: glucosylceramide synthase
GSL: glycosylated sphingolipids
IFN: interferon
KDSR: ketodihydrosphingosine reductase
LCS: lactosylceramide synthase
LPP: lipid phosphate phosphatase
MMR: measles mumps and rubella
NK: natural killer
RENI: renal endocrine neurological immune
S1P: sphingosine-1-phosphate
SPL: sphingosine phosphate lyase
SPLIS: sphingosine phosphate lyase insufficiency syndrome
SPT: serine palmitoyltransferase
TMP/SMX: trimethoprim/sulfamethoxazole
TREC: T cell receptor excision circle.
